# mHealth Systems Need a Privacy-by-Design Approach: Commentary on “Federated Machine Learning, Privacy-Enhancing Technologies, and Data Protection Laws in Medical Research: Scoping Review”

**DOI:** 10.2196/46700

**Published:** 2023-03-30

**Authors:** Ambuj Tewari

**Affiliations:** 1 Department of Statistics University of Michigan Ann Arbor, MI United States; 2 Department of Electrical Engineering and Computer Science University of Michigan Ann Arbor, MI United States

**Keywords:** mHealth, differential privacy, private synthetic data, federated learning, data protection regulation, data protection by design, privacy protection, General Data Protection Regulation, GDPR compliance, privacy-preserving technologies, secure multiparty computation, multiparty computation, machine learning, privacy

## Abstract

Brauneck and colleagues have combined technical and legal perspectives in their timely and valuable paper “Federated Machine Learning, Privacy-Enhancing Technologies, and Data Protection Laws in Medical Research: Scoping Review.” Researchers who design mobile health (mHealth) systems must adopt the same privacy-by-design approach that privacy regulations (eg, General Data Protection Regulation) do. In order to do this successfully, we will have to overcome implementation challenges in privacy-enhancing technologies such as differential privacy. We will also have to pay close attention to emerging technologies such as private synthetic data generation.

## Introduction

Brauneck et al [1] should be congratulated for reviewing privacy-enhancing technologies (PETs) from a legal standpoint. The right to privacy is a fundamental human right, the importance of which in the current digital age cannot be overstated. Protecting this basic human right will need the cooperation of scholars and experts from many disciplines. It is therefore heartening to see legal experts joining hands with technical experts to engage in a thoughtful discussion of how General Data Protection Regulation (GDPR) legislation in the European Union relates to commonly used PETs including federated learning (FL), differential privacy (DP), and secure multiparty computation (SMPC).

The GDPR recognizes that privacy should be a primary design consideration when designing systems that deal with personal data. Privacy is not something to be added on as an afterthought once the system has already been designed. Researchers in the health sciences, especially mobile health (mHealth), are beginning to adopt a “privacy-by-design” mindset. My own group at the University of Michigan and my clinical collaborators have started to seriously study privacy in the context of mHealth [2,3], but much remains to be done.

## Differential Privacy

Brauneck et al [1] correctly point out that FL alone does not sufficiently protect user privacy. This is well known. In fact, the original paper that proposed FL itself pointed out that FL will have to be supplemented with technologies such as DP and SMPC to achieve adequate privacy protection. In this commentary, I will primarily focus on DP. Since I am not a legal expert, my comments will necessarily be from a technical perspective.

DP and its variants have emerged as a leading PET. It has been adopted by technology companies such as Apple and Google. The US Census Bureau also chose it for the 2020 US Census. Calls to revisit foundational statistical theory to incorporate privacy constraints have also formulated the problem using DP [4].

DP has some clear strengths. It is a clear formalism with desirable theoretical properties and increasing software support. However, the epsilon parameter in DP is hard to interpret in the context of specific applications. Its mathematical meaning is precise, but it is often very hard to choose a good value of epsilon to achieve a careful balance between privacy and statistical utility. Researchers have proposed building an “Epsilon Registry” to help the community make sensible implementation choices [5]. More community efforts, especially from the medical informatics community, will be needed to successfully realize the potential of DP.

It is also important to note that recent DP literature is nicely complemented by older statistics literature on statistical disclosure control [6]. It is unlikely that a one-size-fits-all solution will emerge for all data protection scenarios. It is therefore important for system designers to have a broad understanding of available tools. Moreover, old and new tools need to be examined from a legal perspective just as Brauneck et al [1] have done for FL, DP, and SMPC. This is challenging because technology and the law are both undergoing changes. Hopefully, PETs and privacy laws will coevolve so that society will benefit from the ongoing data revolution without threats to the fundamental human right to privacy.

## Private Synthetic Data

Brauneck et al [1] do not mention private synthetic data generation as a PET, but I believe that private synthetic data has tremendous potential for enabling data-driven innovation in health care without sacrificing privacy. The use case the authors considered is one where a data processing workflow (eg, FL) needs to be modified to ensure that it satisfies DP. A different use case is where we simply publish synthetic data that “is similar” to the original sensitive data but which protects user privacy (eg, in the DP sense). This way, downstream data analysts do not have to modify their workflows and can simply work with the synthetic data just as they would with the original data.

However, what does it mean to “be similar” to the original data set? One possibility is that one might hope to preserve correlations between attributes. For a while, it was thought that this could only be done using methods that will be computationally intractable. However, there is recent progress in this area [7], which has renewed interest in the possibility of generating statistically useful synthetic data that nevertheless provably protects user privacy.

## Abbreviations

DP: differential privacy
FL: federated learning
GDPR: General Data Protection Regulation
mHealth: mobile health
PET: privacy-enhancing technology
SMPC: secure multiparty computation
